# CR4 Signaling Contributes to a DC-Driven Enhanced Immune Response Against Complement-Opsonized HIV-1

**DOI:** 10.3389/fimmu.2020.02010

**Published:** 2020-08-14

**Authors:** Marta Bermejo-Jambrina, Michael Blatzer, Paula Jauregui-Onieva, Teodor E. Yordanov, Paul Hörtnagl, Taras Valovka, Lukas A. Huber, Doris Wilflingseder, Wilfried Posch

**Affiliations:** ^1^Institute of Hygiene and Medical Microbiology, Medical University of Innsbruck, Innsbruck, Austria; ^2^Department of Experimental Immunology, Amsterdam Infection and Immunity Institute, Academic Medical Center, University of Amsterdam, Amsterdam, Netherlands; ^3^Experimental Neuropathology Unit, Infection and Epidemiology Department, Institute Pasteur, Paris, France; ^4^Institute of Cell Biology, Biocenter, Medical University of Innsbruck, Innsbruck, Austria; ^5^Central Institute for Blood Transfusion and Immunological Department, Innsbruck, Austria; ^6^Department of Pediatrics I, Medical University of Innsbruck, Innsbruck, Austria

**Keywords:** HIV-1, dendritic cell, complement, CD11c, CD11b

## Abstract

Dendritic cells (DCs) possess intrinsic cellular defense mechanisms to specifically inhibit HIV-1 replication. In turn, HIV-1 has evolved strategies to evade innate immune sensing by DCs resulting in suboptimal maturation and poor antiviral immune responses. We previously showed that complement-opsonized HIV-1 (HIV-C) was able to efficiently infect various DC subsets significantly higher than non-opsonized HIV-1 (HIV) and therefore also mediate a higher antiviral immunity. Thus, complement coating of HIV-1 might play a role with respect to viral control occurring early during infection via modulation of DCs. To determine in detail which complement receptors (CRs) expressed on DCs was responsible for infection and superior pro-inflammatory and antiviral effects, we generated stable deletion mutants for the α-chains of CR3, CD11b, and CR4, CD11c using CRISPR/Cas9 in THP1-derived DCs. We found that CD11c deletion resulted in impaired DC infection as well as antiviral and pro-inflammatory immunity upon exposure to complement-coated HIV-1. In contrast, sole expression of CD11b on DCs shifted the cells to an anti-inflammatory, regulatory DC type. We here illustrated that CR4 comprised of CD11c and CD18 is the major player with respect to DC infection associated with a potent early pro-inflammatory immune response. A more detailed characterization of CR3 and CR4 functions using our powerful tool might open novel avenues for early therapeutic intervention during HIV-1 infection.

## Introduction

Dendritic cells (DCs) play a pivotal role in the defense against invading pathogens, acting as the most potent antigen-presenting cells (APCs) of the innate immune system (1–3). They reside in the peripheral tissue, where they capture antigens and present them to naïve T cells in the lymph nodes. Hence, DCs orchestrate immune responses, serving as critical links between innate and adaptive immunity. DCs are among the first cells to encounter HIV-1 at mucosal sites (2, 4). At the same time, HIV-1 spontaneously activates the classical complement (C-) pathway (5), even in seminal fluid (6), through direct binding of C1q to the viral surface. Therefore, complement-opsonized HIV-1 (HIV-C) accumulates at mucosal sites early during HIV-1 infection (7, 8). HIV-1 poorly replicates in DCs due to the activity of SAMHD1 [Sterile Alpha Motif (SAM) domain and histidine/aspartic acid (HD) domain containing protein 1] and effectively evades DC-mediated antiviral immunity (9). When SAMHD1 restriction of HIV-1 was abrogated by degradation of HIV-2/SIVsm viral protein Vpx, DCs demonstrated a potent type I IFN response, maturation and co-stimulatory function (10). Further, phosphorylation of the T592 residue of SAMHD1 in DCs after exposure to HIV-C overcame this restriction mechanism and initiated an effective antiviral immune response (9). The low-Beside hiding from DC-mediated immunity by low-level infection, the virus additionally exploits DCs as shuttles to promote its own dissemination (11, 12).

In previous studies we demonstrated that HIV-C has the ability to bypass SAMHD1 restriction in DCs, which resulted in more pronounced maturation and significantly higher co-stimulatory capacity compared to DCs exposed to non-opsonized HIV (9).

Additionally, complement coating of HIV-1 further activated highly functional HIV-1 specific cellular immunity as well as pro-inflammatory and type I IFN responses (9, 13, 14). Thus, enhanced DC infection was associated with an increased quality and quantity of virus-specific immune responses (9, 10, 15, 16). We could also show that HIV-C interacts with the abundantly expressed CR3 and CR4 on immature DCs (iDCs), whereas non-opsonized HIV binds via gp120 to DC-SIGN (9, 17). Taken together, these already published results clearly indicate that triggering CR3 and CR4 by HIV-C influences infection of DCs and strongly shapes immunity driven by DCs.

Here, we analyzed in detail the specific roles of CR3 and CR4 in modulating the immune response of HIV-1-infected DCs by generating knock-out (KO) cell lines lacking CD11b, CD11c, and CD18, respectively. For this we performed CRISPR/Cas9 to generate stable and irreversible deletions of these receptors in THP1 monocytes. Furthermore, we optimized the differentiation protocol to generate THP1 derived DCs and to use these THP1-differentiated DCs (THP1-DCs), comprising an iDC phenotype, as an operative model for primary DC infection. After detailed comparison of THP1-DCs with primary DCs at phenotypic and phagocytic properties, we characterized the specific tasks of CR3 and CR4 on THP1-KO DCs with respect to HIV-1 infection and antiviral immune induction using differentially opsonized HIV-1. Here we identified CR4 as potent inducer of early antiviral immunity. Further, the importance of CR3 and CR4 fine-tuning on DCs with respect to controlling viremia during the acute phase of HIV-1 infection by CR4 or down-modulating type I IFNs during chronic phase by CR3 was highlighted.

## Materials and Methods

### Ethics Statement

Written informed consent was obtained from all participating blood donors by the Central Institute for Blood Transfusion and Immunological Department, Innsbruck, Austria. The use of anonymized leftover specimens for research on host/pathogen interactions was approved by the Ethics Committee of the Medical University of Innsbruck (ECS 1166/2018, PI: DW).

### Generation of Human Monocyte-Derived DCs and THP1-DCs

Blood for the monocyte isolation was received by the Central Institute for Blood Transfusion and Immunological Department, Innsbruck, Austria. Briefly, PBMCs (peripheral blood mononuclear cells) were isolated from blood of healthy donors (8, 16) obtained by a density gradient centrifugation using a Ficoll Paque Premium (GE Healthcare) gradient. After washing, CD14^+^ monocytes were isolated from PBMCs using anti-human CD14 Magnetic Beads (BD) – the purity of the isolated cells was at least 98%. Monocytes were stimulated by addition of IL-4 (200 U/ml) and GM-CSF (300 U/ml) for 5 days to generate iDCs, which were used for all further experiments. Non-stimulated iDCs were used as controls for all experiments using DCs. THP1-WT and KO DCs were generated from the respective THP1 cells by addition of IL-4 (200 U/ml), GM-CSF (300 U/ml) and TNF-α (10 ng/ml) for 5 days.

### Genome Editing Using CRISPR/Cas9-Mediated Depletion of CD11b, CD11c, and CD18

For CRISPR/Cas9-mediated depletion, three guide RNA (gRNA) targeting sequences for CD11b, CD11c, and CD18 as depicted in Table 1 were selected using an online prediction tool—CRISPR Design; Zhang Lab (18). Out of the three constructs, only one clone for each target [CD11b (5′-GCCGTAGGTTGGATCCAAACAGG-3′), CD11c (5′-GTAGAGGCCACCCGTTTGGTTGG-3′) and CD18 (5′-TGGCCGGTGTCGCSGCGSSTGG-3′)] was used for further analyses. gRNAs were cloned into a lentiCRISPRv2 vector via *Bsm*BI restriction sites. lentiCRISPRv2 was a gift from F. Zhang (Massachusetts Institute of Technology, Cambridge, MA, United States; Addgene plasmid 52961 (19).

**TABLE 1:** CRISPR/Cas9 gRNA sequences used to produces the THP-1 CD11b KO, THP-1 CD11c KO, and THP-1 CD18 KO.

**Number**	**Target gene**	**Sequence**
1	CD11b exon5	GCCGTAGGTTGGATCCAAACAGG
2	CD11b exon6	TCATCCGCCGAAAGTCATGTGGG
3	CD11b exon6	TTCATCCGCCGAAAGTCATGTGG
4	CD11c exon3	GTAGAGGCCACCCGTTTGGTTGG
5	CD11c exon3	ACTGGTAGAGGCCACCCGTTTGG
6	CD11c exon4	GACATGTTCACGGCCTCCGGGGG
7	CD18 exon3	GCCGGGAATGCATCGAGTCGGGG
8	CD18 exon3	TGCCGGGAATGCATCGAGTCGGG
9	CD18 exon4	TGGCCGGGTGTCGCAGCGAATGG

*Indicated sequences target different exons of the CD11b, CD11c, and CD18 genes. One clone out of the three was used for further analyses.*

### Lentiviral Transduction

Lentiviral plasmids were co-transfected with Lipofectamine LTX (Invitrogen, cat 15338100) together with pMDG, psPAX2 and lentiCRISPRv2 into the HEK293T producer cell line. Supernatants containing viral particles were harvested 48 and 72 h post transfection, filtered using a 0.2 μm filter and directly used to transduce target THP1 cells with 5 μg/ml Polybrene (Sigma-Aldrich, cat TR-1003-G). After 7 days, transduced cells were selected using puromycin (5 μg/ml, Sigma-Aldrich, cat SBR00017). After selection, the depletion efficiency of CD11b, CD11c, and CD18 was analyzed by flow cytometry. Single-cell clones of the specific KO cells were generated after FACS sorting by the Core Facility FACS Sorting at the Medical University of Innsbruck.

### Virus Production

Primary isolates as 92BR030 (subtype B/B, R5-tropic) and the laboratory strain BaL were obtained by the National Institutes of Health AIDS (available through World Health Organization depositories). Virus was propagated in PHA-L and IL-2 stimulated PBMCs. 93BR020 (subtype B/F, X4/R5-tropic) and the laboratory strain NL43 both from National Institutes of Health AIDS (available through World Health Organization depositories) were produced in the M8166 cell line. HEK293T cells were transfected with YU- 2-, and R9Bal (kindly provided by Prof. Thomas Hope, Northwestern University) plasmids using the CaCl_2_ method (9). Vpx expression construct pcDNA3.1Vpx SIVmac239-Myc was used to obtain Vpx-carrying HIV virus preparations (20). Viral supernatants were collected on several days post infection (dpi) and cleared by filtration through 0.22 μm pore-size filters and concentrated by ultracentrifugation at 20,000 rpm for 90 min at 4°C (Beckham Coulter). The virus pellet was re-suspended in RPMI1640 without supplements and stored in small aliquots at −80°C to avoid multiple thawing. One aliquot was taken to determine the virus concentration by p24 ELISA (21) and the 50% tissue culture infective dose of the viral stock.

### Opsonization of Viral Stocks

To mimic opsonization *in vitro*, purified HIV-1 and VLP stocks were incubated for 1 h at 37°C with human complement (C) serum (Quidel) in a 1:10 dilution. As negative control the virus was incubated under the same conditions in commercially available C3-deficient serum (Sigma) or in culture medium. After opsonization, the virus was thoroughly washed to remove unbound components, pelleted by ultracentrifugation (20,000 rpm/90 min/4°C), re-suspended in culture medium without supplements and virus concentrations were determined using p24 ELISA. The opsonization pattern was analyzed using a virus capture assay (VCA) described below.

### Virus Capture Assay

The opsonization pattern was determined by virus capture assay (VCA) as described (8). Briefly, 96-well high-binding plates were coated with anti-human C3c, C3d, or IgG antibodies. Mouse IgG antibody was used as a control for background binding. Plates were then incubated overnight at 4°C with the differentially opsonized virus preparations (10 ng p24/well) at 4°C. After extensive washing, virus was lysed and p24 ELISA was performed to confirm the opsonization pattern.

### p24 ELISA

p24 ELISA was performed as described (21). Antibodies used for p24 ELISA were kindly provided by Polymun Scientific, Vienna, Austria.

### DC Infection

Cells were infected in triplicates using differentially opsonized HIV-1 as described before (8, 17). Briefly, cells (1 × 10^5^/100 μl) were incubated for 3 h with HIV or HIV-C (25 ng p24/ml) or left uninfected and virus concentrations from supernatants were measured on several dpi. To confirm productive infection by HIV-1 and not cell-associated virus, we thoroughly washed the cells after overnight incubation with different viruses and cultured the cells at 37°C/5% CO_2_. By ELISA we measured the p24 concentrations of the supernatants following spinning down the plate to pellet cells on several dpi. The following antibodies were used for blocking experiments (all anti-human): LEAF purified CD11b-Antibody (Biolegend, San Diego, CA, United States), LEAF purified CD11c-Antibody (Biolegend, San Diego, CA, Untied States).

### Immunoblot Analyses of Phosphorylated Proteins

THP1-DCs were starved in RPMI 1640 containing 0.5% FCS and 1% L-Glutamine for 3 h. Starving of cells was performed to set their phosphorylation to background levels. Following starvation, THP1-DCs were incubated with the differentially opsonized HIV-1 particles. After 4 h co-incubation, cells were lysed with RIPA Buffer (Sigma-Aldrich) containing protease and phosphates inhibitors and EDTA (Thermo Fisher Scientific) for 20 min at 4°C. The protein content was determined by BCA (Thermo Fisher Scientific). Lysates were separated using 10% SDS-PAGE gels, transferred to PVDF membranes and incubated with anti-human a-tubulin as loading control as well as anti-human phospho-IRF3 (1:1000, Cell Signaling Technology) and developed with the Lass 4000 Image Quant. For this, the peak values of the target protein were divided by the peak values of the loading control before doing a relative comparison. Quantification was performed using values from three to six different experiments.

### Relative Quantification by Real-Time RT-PCR

THP1-DCs (WT and KOs) were infected with the differentially opsonized HIV-1 particles at different time-points from 1–12 h at 37°C with a p24 concentration of 350 ng/mL for 0.5 × 10^6^ cells. Cells were lysed with RLT Buffer (Qiagen) and total RNA was purified according to the manufacturer’s instructions. RNA was then quantified (NanoVue) and reverse transcribed into cDNA (iScript Reverse Transcription Supermix for RT-qPCR, BioRad). The cDNA was then used for multiplex qPCR (iQ Multiplex Powermix, BioRad) amplification, using PrimePCR^TM^ Probes for IL-10, IL-6, IL-1B, and IL23A (all from BioRad Laboratories). The RT-qPCR was run in the BioRad CFX96 Real Time PCR System. The cycling conditions were as follows: 3 min at 95°C, 44 cycles: 15 s at 95°C, 60 s at 60°C. For mRNA expression of IFNB1 real-time PCR using Sybr green qPCR (EvaGreen, BioRad) amplification and gene-specific primer pairs (BioRad) were used. The cycling conditions were: 30 s at 95°C, 39 cycles: 5 s at 95°C, 10 s at 60°C with a melt curve 65–95°C with an increment of 0.5°C for 5 s. A GAPDH (human) PCR using specific primer/probe pairs (BioRad) served as internal control to quantify the relative gene expression of target genes. Data were analyzed with the BioRad CFX Manager Software (ΔΔCT method) and values were exported to GraphPad Prism.

### Cytokine Analyses by ELISA

THP1-DCs were plated in a 12-well tissue culture-plate at 0.5 × 10^6^ cells/well. Cells were infected with R5-tropic virus (R9Bal) for 12, 24, and 48 h. Supernatants were collected and inactivated with Igepal 5% (1:2). The amounts of IL-1β were measured by ELISA (eBioscience).

### Multicolor FACS Analyses

Differentiation of THP1 into DCs exposed to cytokine cocktail (IL-4, GM-CSF, TNF-α) was analyzed by using anti-human CD11b-PE, CD11c-AlexaFluor488, CD18-APC, HLA-ABC-PerCP/Cy5.5, HLA-DR-APC-Cy7, DC-SIGN-PE, CD86-FITC, CD83-APC, CD1a-FITC, CD4-APC, CCR-PerCP/Cy5.5 and CXCR4-PE as described (16) on a FACS Verse flow cytometer (BD Biosciences). Data were analyzed using FACS DIVA software (BD Biosciences).

### Statistical Analysis

Differences were analyzed by using GraphPad Prism software (GraphPad Software Inc.) and one-way ANOVA with Bonferroni post-test for multiple comparisons or Unpaired Student’s t test depending on the analyses performed.

## Results

### WT THP1-DCs and KO THP1-DCs Resemble Primary DCs Regarding Their Phenotypic and Phagocytic Capacities

To characterize CR3 and CR4 with respect to DC modulation upon exposure to differentially opsonized HIV-1, we generated CD11b-, CD11c-, and CD18 KO THP1-DCs. Since THP1 monocytes constitute an immortalized cell line and are of tumorigenic origin derived from the peripheral blood of a one-year-old male with acute monocytic leukemia, we wanted to make sure to generate an appropriate model for KO DCs. Therefore, we first characterized by multi-parameter flow cytometric analyses in detail WT-THP1 cells after optimized differentiation to DCs for their expression of characteristic DC markers, CR3 and CR4 and HIV-1 receptor and co-receptors CD4, CCR5, and CXCR4.

**FIGURE 1:**
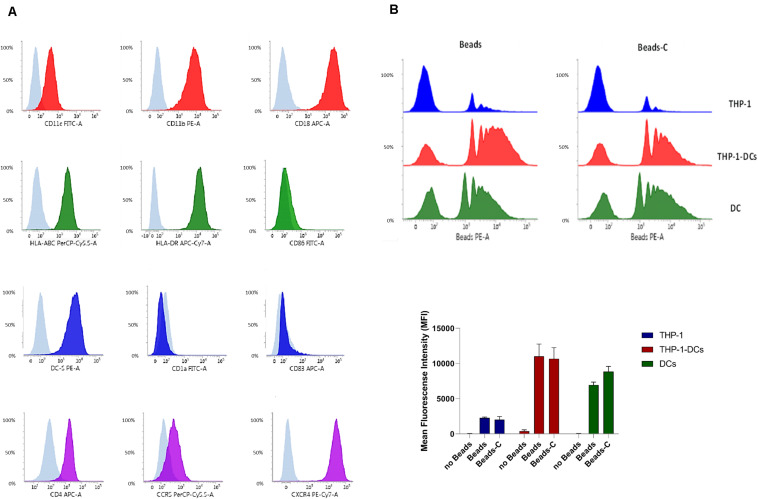
THP1 monocytes can be differentiated into functional DCs. **(A)** WT THP1 cells were differentiated into DCs and expression of characteristic DC markers CD11c, CD11b, CD18, HLA-ABC, HLA-DR, CD86, DC-SIGN, CD1a, CD83, and HIV-1 receptors CD4, CCR5, and CXCR4 was analyzed using flow cytometry. A representative flow cytometry analysis is shown out of five independent experiments. **(B)** Phagocytosis of non- and complement-opsonized Beads (Beads, Beads-C) was analyzed in WT THP1 monocytes (blue), WT THP1-DCs (red), and monocyte-derived DCs (green). THP1-DCs (red) exerted similar phagocytosis capacities with respect to Beads and Beads-C compared to monocyte-derived DCs (green), while THP1 monocytes had a very low phagocytic activity (blue). Panel **(B)** illustrates a FACS analysis from one representative experiment (upper panel) and data from three independent experiments are summarized in panel **(B)** (lower panel).

We found that THP1-DCs differentiated *in vitro* into a functional DC-like phenotype (Figure 1A and Supplementary Figure S1>monocyte-derived iDCs, moDCs), expressing high levels of both CRs, CR3, and CR4, as analyzed by the expression of CD11b, CD11c, and CD18. HLA-ABC, HLA-DR, and DC-SIGN were also found to be expressed on WT-THP1 DCs, and also on moDCs as illustrated by Posch et al. (9) and in Supplementary Figure S1. No expression of CD1a was detected. Importantly, low levels of CD83 and CD86 were indicative of an iDC state and displayed a mature phenotype upon LPS stimulation (not shown). The profile of characteristic markers CD11b, CD11c, CD18, DC-SIGN, CD83, CD86, CD4, CXCR4, and HLA-DR on immature moDCs is illustrated in Supplementary Figure S1. Entry of HIV-1 into target cells requires formation of a complex between the viral envelope protein gp120, the primary receptor CD4 and a chemokine co-receptor (CCR5, CXCR4). We found that THP1-DCs expressed similar amounts of CD4, CCR5, and CXCR4 as primary DCs.

THP1-DCs further illustrated a similar phagocytic capacity as their primary counterparts (Figure 1B). Phagocytosis of non- and complement-opsonized beads (Beads, Beads-C) was low in THP1 monocytes, while WT THP1-DCs demonstrated a similar phagocytosis of Beads/Beads-C compared to monocyte-derived DCs.

**FIGURE 2:**
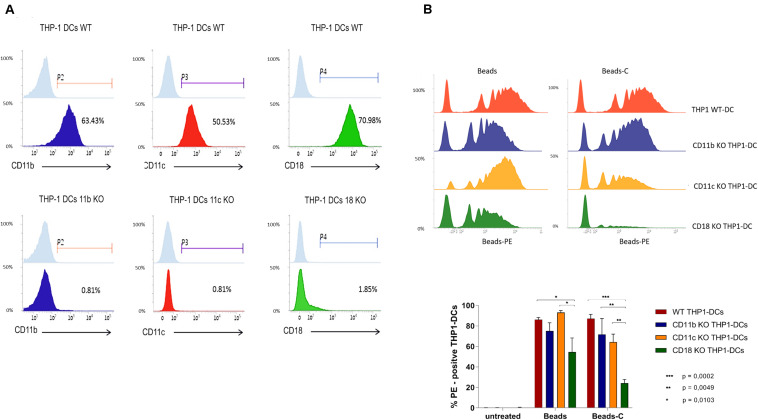
CD11b-, CD11c-, and CD18 KO THP1 DCs comprise a novel tool to decrypt distinct functions of CR3 and CR4. **(A)** CD11b, CD11c, or CD18 were stably deleted by using the CRISPR/Cas9 genome editing technology. Top panel: WT THP1 cells differentiated to DCs were used as controls for monitoring expression of CD11b (blue), CD11c (red), and CD18 (green). Bottom panel: CD11b KO THP1-DCs do not express CD11b (blue), CD11c KO THP1-DCs are devoid of CD11c (red), and no CD18 is expressed on CD18 KO THP1-DCs (green). A representative flow cytometric analysis from one out of five experiments is illustrated. **(B)** Phagocytosis of C-opsonized Beads is hampered in CD11c- (yellow) and CD18 KO (green) THP1-DCs compared to WT (red) and CD11b KO (blue) THP1-DCs. Lowest phagocytosis using non-opsonized Beads was monitored in CD18 KO THP1 DCs (green). A representative flow cytometric analysis out of three independent experiments is depicted.

Next we investigated the expression of CD11b, CD11c, and CD18 on KO THP1-DCs generated by CRISPR-Cas9 technology. CD11b expression on single-cell clones of CD11b KO THP1-DCs was reduced to 0.81% compared to 63.43% on WT THP1-DCs (Figure 2A). CD11c was only slightly affected on CD11b KO THP1-DCs (Supplementary Figure S2). Expression of CD11c on CD11c KO THP1-DCs was also reduced from 50.53% on WT THP1-DCs to 0.81% (Figure 2A), while CD11b was expressed on CD11c KO THP1-DCs (Supplementary Figure S2). In contrast, CD18 KO resulted in significant down-modulation of CD11b as well as CD11c (Supplementary Figure S2) and in addition, also CD11a disappeared from the surface of CD18 KO THP1-DCs, but not on CD11b- and CD11c KO THP1-DCs (Supplementary Figure S3). Phagocytosis of the various KO THP1-DCs revealed that in CD11b KO cells the levels of Beads or Beads-C internalized slightly decreased compared to WT THP1-DCs (Figure 2B), while CD11c KO had a highly decreased phagocytosis of Beads-C, but not Beads (Figure 2B). KO CD18 severely reduced the amounts of Beads ingested and completely abrogated internalization of Beads-C (Figure 2B). We further focused on CD11b- and CD11c KO THP1-DCs throughout the manuscript and CD18 KO-THP1 DCs were used in some experiments as controls. Phenotypic and phagocytic characterization of WT and KO THP1-DCs revealed these cells as a good DC model to study distinct roles of CR3 and CR4 during the very early steps of HIV-1 infection.

### CR4 Plays a Major Role With Respect to HIV-C Infection of DCs

**FIGURE 3:**
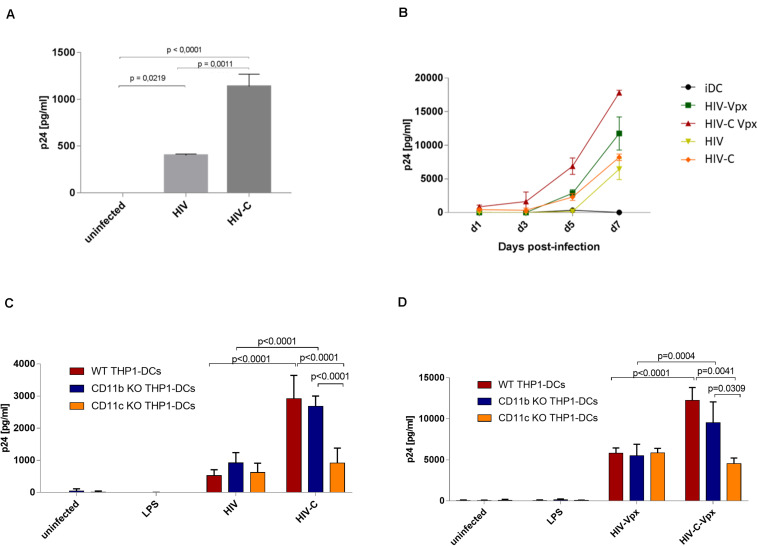
Complement-opsonized HIV-1 effectively infects WT- and CD11b KO THP1-DCs, while productive HIV-1 infection is impaired in CD11c KO THP1-DCs. **(A,B)** Upon infection of WT THP1 DCs with HIV or HIV-C (25 ng p24/ml) a significantly enhanced productive infection was monitored using HIV-C. Means ± SD from three independent experiments in duplicates are depicted in **(A)**. Differences were analyzed using Unpaired Student’s *t*-test. In **(B)** a time-course of WT THP1-DC infection exposed to HIV (lime), HIV-C (orange), HIV-Vpx (green), and HIV-Vpx-C (red) is illustrated. Experiments were repeated three times in duplicates. **(C,D)** WT- (red), CD11b KO (blue), and CD11c KO (yellow) THP1-DCs were infected with HIV or HIV-C (C) and HIV-Vpx or HIV-C-Vpx. Uninfected and LPS-incubated THP1-DCs served as controls. Infection experiments were performed in three independent experiments performed in triplicates. Differences were analyzed using GraphPad Prism and one-way ANOVA with Bonferroni post-test.

Efficient antiviral T cell responses are initiated when DCs are productively infected by HIV-1 after their resistance to infection is bypassed (10, 13). In contrast, the inability of DCs to become infected is supposed to be an evasion strategy for HIV-1 survival. As previously shown by our group and also herein in Supplementary Figure S4, DCs are efficiently infected by complement-opsonized HIV-1 (HIV-C), while only low-level productive infection was mediated by HIV-1 (HIV) (8, 9). To unravel the specific roles of CR3 and CR4 with respect to productive DC infection, we first analyzed infection of WT THP1-DCs after exposure to HIV or HIV-C. As previously demonstrated, virus concentrations of WT THP1-DCs were similar to the ones obtained in primary DCs (13). Thus, low-level productive infection was only monitored in WT THP1-DCs exposed to HIV, whereas infection was significantly enhanced using HIV-C (Figure 3A). Non-infected and therefore immature THP1-DCs were used as negative controls (Figure 3A, uninfected or UI). Consistently with Posch et al. (9) using monocyte-derived DCs (moDCs), we could also illustrate that infection of WT THP1-DCs with Vpx-carrying HIV illustrated a similar pattern compared to HIV-C by enhancing productive infection compared to non-opsonized HIV. In addition, complement opsonization of HIV-Vpx (HIV-C Vpx) improved infection even more (Figure 3B). Since same infection patterns could be displayed in THP1-DCs, moDCs and BDCA1^+^ DCs (9), we continued the next steps using WT THP1-DCs and their CD11b- and CD11c KO THP1-DCs counterparts to characterize in detail the specific roles of CR3 and CR4 during HIV-1 infection. We found that infection of CD11b- or CD11c KO THP1-DCs with non-opsonized HIV (HIV, Figure 3C) or non-opsonized Vpx-carrying HIV (HIV-Vpx, Figure 3D) was similar to infection levels of WT THP1 DCs. Vpx-carrying HIV-1 mediated a more than fivefold enhanced infection compared to the low-level productive infection induced by non-opsonized HIV-1. In contrast, infection of WT- or CD11b KO THP1-DCs with complement-opsonized HIV (HIV-C, Figure 3C) was significantly enhanced similar to moDCs (Supplementary Figure S4A). This was also the case for complement-opsonized Vpx-carrying HIV (HIV-C Vpx, Figure 3D).

In contrast, CD11c KO-THP1 DCs showed similar p24 levels between HIV and HIV-C or HIV-Vpx and HIV-C Vpx (Figures 3C,D, yellow), and productive infection using HIV-C or HIV-C Vpx was significantly reduced when compared to CD11b KO or WT THP1-DCs (Figures 3C,D, HIV-C, yellow vs. blue and red bars). Uninfected iDCs and LPS-exposed THP1-DCs were used as controls. Using blocking antibodies against CD11b and CD11c and moDCs revealed similar results as the THP1 KO DC models. While CD11b blocking significantly enhanced productive DC infection upon exposure to HIV-C, blocking CD11c significantly decreased productive infection as seen also in CD11c KO THP1 DCs (Supplementary Figure S4B). This reduction was in part rescued when combining the CD11b/CD11c blocking Abs (Supplementary Figure S4B), highlighting the cross-talk of these two receptors.

These experiments demonstrated that THP1-DCs represent a valid model for DC infection, due to the similar infection kinetics observed in WT THP1-DCs compared to primary DCs and also because complement opsonization of HIV-1 significantly enhanced productive DC infection. Furthermore, our data revealed that CR3 is not involved in infection of DCs by HIV-C, since CD11b KO THP1-DCs or CD11b blocking using a blocking anti-human CD11b mAb showed a significant HIV-C-mediated enhancement of DC infection (Figure 3 and Supplementary Figure S4B). In contrast, deleting CD11c (CR4) had a severe effect on DC infection, which caused a low-level productive DC infection with complement-opsonized HIV-1, comparable to the low-level infection observed using non-opsonized HIV-1 (Figure 3 and Supplementary Figure S4B). To summarize, abrogation of CR3 does not impact productive infection with HIV-C, while CR4 KO results in low-level DC infection comparable to HIV.

### CR4 KO Diminishes Antiviral Signaling Pathways and Mediates an Anti-inflammatory DC Type

To determine, whether CR4 KO also impacts the antiviral and inflammatory DC profile induced by HIV-C, we studied antiviral signaling pathway IRF3 and type I IFN expression, IL-1β production and mRNA level expression of IL6, IL10, and IL23A.

**FIGURE 4:**
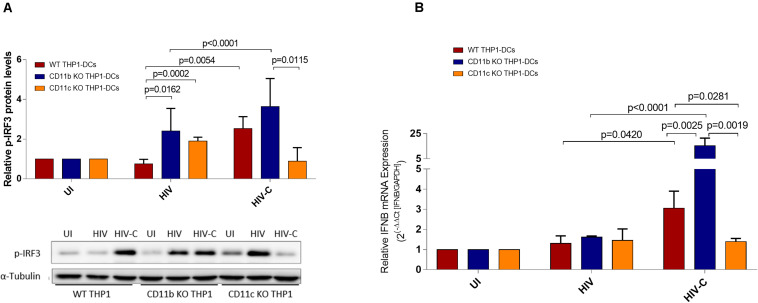
Antiviral signaling pathways are impeded in CD11c-deleted THP1 DCs. **(A)** Relative p- p-IRF3 protein levels were assessed in WT- (red), CD11b KO (blue), and CD11c KO (yellow) THP1-DCs. Experiments were repeated four times independently and a summary as well as a representative immunoblot are depicted. UI, uninfected. **(B)** RT-PCR analyses of type I IFN (IFNB) levels in WT-, CD11b KO-, and CD11c KO-THP1 DCs after infection with HIV or HIV-C (BaL, YU-2). Non-infected iDC were used as controls. Data are mean ± SD from four different donors in duplicates. Differences were analyzed by using one-way ANOVA with Bonferroni post-test.

Antiviral signaling pathways involving TANK Binding Kinase 1 (TBK1) and Interferon regulatory factor 3 (IRF3) are associated with induction of an early type I IFN response. Upon analyzing IRF3 phosphorylation after exposure of DCs to HIV or HIV-C (Figure 4A), we found significantly increased activation levels in CD11b KO THP1-DCs upon stimulation with both, HIV- and HIV-C (Figure 4A). Stimulation of CD11c KO THP1-DCs with HIV, too, increased IRF3 phosphorylation, while in HIV-C-exposed CD11c KO THP1-DCs the levels were significantly decreased compared to CD11b KO THP1-DCs and reduced in comparison to WT THP1-DCs (Figure 4A). This enhanced activation of pIRF3 in CD11b KO THP1-DCs by HIV-C was further associated with a significantly increased expression of type I interferon IFNB, but not in case of HIV-exposed DCs (Figure 4B). In contrast, CD11c KO cells did not show any change on IFNB mRNA expression levels compared to WT- and CD11b KO-THP1 DCs (Figure 4B).

**FIGURE 5:**
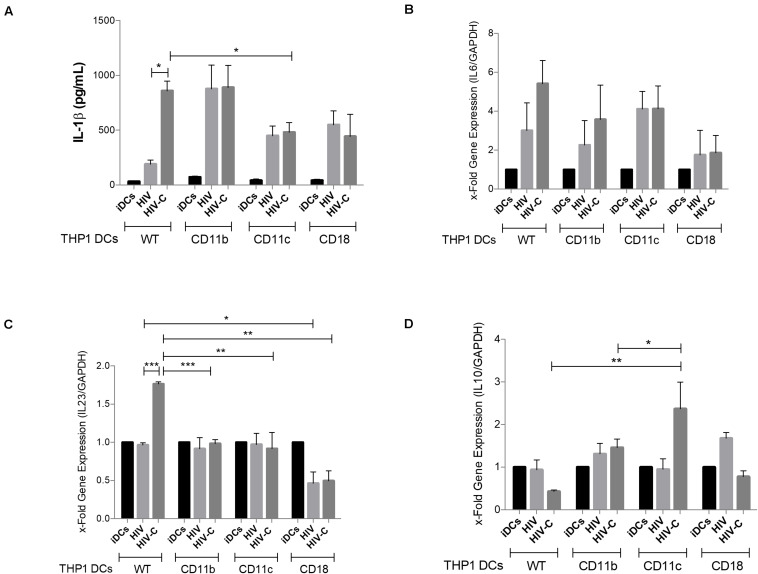
CD11c deletion significantly modifies the cytokine profile by decreasing IL-1β production and IL23 expression, while up-regulating IL10 levels. IL-1β production **(A)**, IL6 **(B)**, IL23A **(C)**, and IL10 **(D)** expression levels were analyzed in WT-, CD11b KO-, CD11c KO-, and CD18 KO THP1-DCs as indicated following infection with HIV or HIV-C. UI cells served as controls. Experiments were repeated thrice in duplicates and RT-PCR analyses were performed. Differences were analyzed by using GraphPad Prism software and one-way ANOVA with Bonferroni post-test. *p < 0.01, **p < 0.001, ***p < 0.0001.

Next, we studied pro-inflammatory cytokine induction as measured by IL-1β production and IL6, IL10, and IL23A mRNA levels, since in monocyte- and blood-derived DCs we previously found that HIV-C significantly increased production of Th17-polarizing cytokines, such as IL-1β, IL-6, and IL-23, while IL-10 expression was even decreased (14). Strikingly, we found a significantly increased IL-1β secretion in HIV-C-exposed WT THP1-DCs compared to HIV-loaded or iDCs (Figure 5A, WT), corroborating what has already been published in primary DCs. CD11b KO THP1-DCs mediated an augmented IL-1β production, upon exposure to non-opsonized HIV, similar to HIV-C-loaded WT- and CD11b KO THP1-DCs (Figure 5A, CD11b and WT). In contrast, CR4 KO significantly decreased IL-1β levels secreted in HIV-C-exposed CD11c KO THP1-DCs (Figure 5A, CD11c). IL6 and IL23A mRNA expression were significantly enhanced in WT THP1-DCs upon exposure to HIV-C as described in primary DCs (14). In case of CD11c KO THP1-DCs, IL23A was significantly reduced in HIV-C-exposed DCs to levels mediated by HIV (Figure 5C). In addition, IL6 mRNA expression was reduced, although not significantly, as for CD11c KO THP1-DCs (Figure 5B). A different picture was observed for IL-10 expression, since this cytokine was expressed at similar levels in all treatments (iDCs, HIV, HIV-C) using WT-, CD11b-, and CD18 KO-THP1 DCs (Figure 5D, WT, CD11b). In contrast, CD11c KO-THP1 DCs significantly increased expression of this anti-inflammatory cytokine upon exposure to HIV-C, but not HIV (Figure 5D, CD11c). Thereby, abolishing CD11c on DCs diminishes the antiviral profile in DCs, while IL-10-producing DCs (DC-10) were induced by knocking out CR4. These data point to a role of CR3 (CD11b/CD18) as regulator of dampening immune responses and CR4 (CD11c/CD18) as inducer of pro-inflammatory and antiviral immune responses.

## Discussion

Integrins are crucial components linking intra- and extracellular environments and thereby coordinating vital features of cellular behavior, such as adhesion, cell contact formation, signaling, immune activation. Among an array of PRRs, DCs abundantly express integrin receptors CR3 (CD11b/CD18) and CR4 (CD11c/CD18), composed of α-M (CD11b) or α-X (CD11c) and the common β2-subunit CD18. Emerging evidence suggests that integrins play an important role in immune activation and inflammation. We recently showed that complement-opsonized HIV-1 (HIV-C) overcomes restriction in DCs by efficiently activating SAMHD1 phosphorylation, and this was associated with a higher DC maturation and co-stimulatory potential, aberrant type I interferon and signaling as well as a stronger induction of pro-inflammatory and cellular immune responses (9, 14). This was not the case for non-opsonized HIV-1 (HIV), which was restricted by SAMHD1 in DCs. Therefore, we here defined in more detail the involvement of the α integrins CD11b and CD11c in this increased DC activation during HIV-1 infection, when the virus was C-opsonized. With this purpose, we generated stable CD11b- and CD11c-KO THP1 DCs using CRISPR/Cas9 technology and characterized them in detail with respect to their similarity to primary monocyte-derived DCs.

Coating of HIV-1 with complement (C-) fragments and binding of HIV-C to CRs might contribute to protecting virus particles from immediate and extensive degradation in intracellular compartments as illustrated for non-opsonized HIV-1 (22). We and others (8, 9, 17, 23, 24) found a significant enhancement of productive DC infection by HIV-C associated with the above mentioned improved antiviral immune responses as well as an adjuvant role with respect to induction of HIV-specific CTLs. This C-mediated, significantly enhanced productive HIV-1 infection was also detected in WT THP1-DCs. After detailed evaluation of WT THP1-DCs on additional characteristics exerted by primary DCs, we proved this cell line to be a suitable model, since similar expression of specific receptors were confirmed. In addition, WT THP1-DCs showed same HIV-1 infection kinetics as primary DCs, since HIV-1 subverts complement for productive infection compared to non-opsonized HIV-1 (9, 13). This complement-mediated enhancement in DC infection was further confirmed using Vpx-carrying HIV and HIV-C Vpx preparations. Productive infection of primary DCs was shown to be limited due to the restriction of SAMHD1, which is not counteracted by non-opsonized HIV-1. However, our group previously illustrated that bypassing SAMHD1 by phosphorylation through HIV-C in iDCs significantly enhanced productive HIV-1 infection and subsequent antiviral humoral and cellular immunity *in vitro* (9). Despite significantly higher SAMHD1 levels in THP1 monocytes (11), HIV-C- and Vpx-mediated effects were also seen in WT THP1-DCs, which proves them to be a valuable model for studying functions of CR3 and CR4 in relation to HIV-1 infection in more detail.

To characterize the KO CD11b and CD11c cells, we infected WT-, CD11b-, and CD11c KO-THP1 DCs using HIV and HIV-C. In CD11b KO-THP1 DCs, HIV-C mediated a significantly enhanced productive infection similar to primary DCs and WT-THP1 DCs. However, the considerably augmented productive infection was lost in CD11c KO-THP1 DCs or upon blocking CD11c on moDCs and infection levels were comparable to the low-level productive infection mediated by HIV. Complement-mediated effects in presence of Vpx were also lost in CD11c KO THP1-DCs only, but not in CD11b KO THP1-DCs. Similar results to CD11c KO THP1-DCs were observed in CD18 KO cells, devoid of both CR3 and CR4.

Our data represent the first evidence of the major role of CR4 in DC infection with complement-opsonized HIV-1, and is in controversy to findings from Tjomsland et al. (25), who illustrated that blocking CR3 significantly decreased infection of emigrating DCs from cervical mucosal tissues. These authors used a combination of blocking antibodies against CD11b and CD18, which could cause CR3 and CR4 blocking due to the shared integrin beta chain-2 (CD18) of both CRs. Thus, effects seen in this study might not rely on CR3 blocking, but probably on blocking CR4-mediated signaling.

IRF3 and IRF7 are the main regulators of type I IFN expression (26). IRF3, localized in the cytoplasm in a latent form, gets activated by phosphorylation via TBK1 or IKKε and translocates to the nucleus. Once in the nucleus, IRF3 dimerizes with NFκB and activating transcription factor 2 (ATF2)–c-jun to recruit CREB-binding protein to the IFNB promoter to form a functional beta interferon “enhanceosome” (27). We found this IRF3/NFκB “enhanceosome” also in primary DCs exposed to HIV-C (9) and within this study we unraveled the CR responsible for this axis using the CD11b/CD11c KO THP1-DCs. In line with the results obtained from the infection analyses, we detected that the p-IRF3 signaling is significantly disrupted upon CD11c KO, but intact in WT- and CD11b KO-THP1 DCs. Disturbance of IRF3 activation was associated with a significantly impaired *IFNB* mRNA level expression in CD11c KO THP1-DCs. These results confirm the findings, that abolishment of IFN-β was observed in DCs deficient of *Irf3* upon LPS stimulation or significantly impaired upon Poly(I:C) treatment (28) and also upon virus infection of *Irf3^–/–^* mouse embryonic fibroblasts (MEFs) (29). Additionally, these activated cascades contribute to the type I IFN positive feedback loop. We observed in WT THP1-DCs that type I *IFNB* mRNA significantly increased in HIV-C infection, leading to a better antiviral response. These findings are consistent with our other study (9) finding elevated levels of *IFNB* mRNA and ISGs in HIV-C exposed primary DCs. Our findings differ from results published by Ellegård et al. (30), who showed that complement opsonization of HIV-1 resulted in a decreased antiviral immune response in DCs. In contrast to that finding, the authors illustrated in line with our data, that DCs are infected to significantly higher levels when the virus was complement-opsonized (30, 31). Furthermore, they illustrated considerably enhanced IRF3 activation in DCs upon HIV-C treatment, which point to induction of an efficient antiviral immune response by DCs. Nonetheless the authors concluded that complement-opsonized HIV dampens immune responses via DCs compared to non-opsonized HIV-1. In contrast, we found, enhanced productive infection of DCs exposed to HIV-C also increases IRF3 activation and phosphorylation of TBK1 only in WT-THP1-DCs and CD11b KO THP1-DCs, but not CD11c KO THP1-DCs. In addition, CD11b KO resulted in an overshooting type I IFN response.

Since CD11b plays a key role in phagocytosis of apoptotic cells, ligation of this member of the heterodimeric β2 integrin family results in production of anti-inflammatory cytokines such as TGF-β or IL-10 (32). At the same time ligation of CD11b negatively regulates pro-inflammatory signals via e.g., TLRs or FcγR (33–35). Our data confirm this anti-inflammatory, IL-10-inducing role of CD11b, which does not only seem to negatively regulate TLR or FcγR signaling pathways (32), but also signaling pathways initiated via CD11c. A role for anti-inflammatory signaling via CD11b was also observed in CD11c KO THP1-DCs that solely express CR3, since in these cells significantly increased IL-10 expression levels were detected similar to DC-10, a human subset of tolerogenic DCs endowed with the ability to spontaneously release IL-10 as described by Comi et al. (36).

Our results nicely reflect the distinct roles of CD11b and CD11c with respect to inflammatory or antiviral host responses, but also point to the importance of balanced levels regarding either elevated, overshooting induction of antiviral signaling pathways (CD11b KO) or dampening via pIRF3/IFNB reduction and IL-10 induction (CD11c KO). Our results in WT- and CD11b KO THP1-DCs showed an enhanced antiviral type I IFN signaling pathway, comparable to the one seen in primary DCs, e.g., BDCA1^+^ DCs (9). The contradictory data seen in our experiments compared to Ellegard et al. (30) might rely on diverse monocyte isolation and differentiation protocols or cell sources to work on and thereby differential expression levels of either CR3 or CR4 on generated DCs.

Our data suggest an important and distinct role for β2 integrins, CR3 and CR4, in myeloid cells. Beyond initial binding of complement-opsonized particles, myeloid cells encounter ligands within the extracellular matrix while en route to their intended targets. Here, these ligands are modified by local inflammatory mediators (37). Dependent on interaction with either CR3 or CR4, inflammatory cytokine production is restricted to minimize damage of the host via CD11b, while CD11c seems to take action with respect to efficient antiviral immune responses in a type I interferon autocrine-paracrine manner. Cooperation of the NFκB-dependent pathway leading to inflammatory cytokine secretion and the IFN-dependent pathway mediating type I IFN and ISGs was also described upon TLR7/8 triggering in DCs (38) and this type I IFN autocrine-paracrine loop seems to also play an important role in CR4-signaling, which has to be confirmed in more detail. Nevertheless, specific targeting of either CD11b or CD11c might be an innovative tool to regulate pro- and anti-inflammatory processes during infectious diseases such as HIV-1.

## Data Availability Statement

All datasets presented in this study are included in the article/Supplementary Material.

## Ethics Statement

The Ethics Committee of the Medical University of Innsbruck approved the study. The study number is ECS 1166/2018, and the PI is the corresponding author DW. The patients/participants provided their written informed consent to participate in this study.

## Author Contributions

DW and WP: conceptualization and funding acquisition. MB-J, MB, PJ-O, TY, TV, LH, WP, and DW: investigation. DW, WP, and MB-J: writing – original draft. DW, MB-J, MB, TV, LH, and WP: writing – review and editing. DW: project administration. All authors contributed to the article and approved the submitted version.

## Conflict of Interest

The authors declare that the research was conducted in the absence of any commercial or financial relationships that could be construed as a potential conflict of interest.
