# Supplementary material for: CR4 Signaling Contributes to a DC-Driven Enhanced Immune Response Against Complement-Opsonized HIV-1
Source: Front Immunol. 2020 Aug 14;11:2010. doi: 10.3389/fimmu.2020.02010 (PMC7457048; doi:10.3389/fimmu.2020.02010)
Supplement: Supplementary file 1 [file Data_Sheet_1.docx]

**Supplementary Figures**

**Supplementary Figure S1 Flow cytometric analysis of CD11b, CD11c, CD18, DC-SIGN, CD83, CD86 and HLA-DR on monocyte-derived DCs (moDCs).** CD11b, CD11c, CD18, DC-SIGN and HLA-DR are highly expressed on monocyte-derived iDCs, while only low expression of markers of mature DCs CD83 and CD86 were monitored.

**Supplementary Figure S2** **Flow cytometric analysis of CD11b, CD11c and CD18 on WT, CD11b, CD11c- and CD18 KO THP1-DCs.** The specific deletion of either CD11b, CD11c or CD18 is illustrated herein and highlights the necessity of CD18 with respect to CD11b and CD11c expression. FACS analyses were repeated four times.

**Supplementary Figure S3 Flow cytometric analysis of CD11a on WT, CD11b-, CD11c- and CD18 KO THP1-DCs.** Expression of CD11a was monitored on CD11b-, CD11c and CD18 KO THP1-DCs and compared to WT THP1 DCs. FACS analyses were repeated four times.

**Supplementary Figure S4. Infection of moDCs by HIV-C is mediated via CR4.** HIV-C induced a significantly enhanced productive infection of moDCs compared to low-level infection of HIV-exposed DCs **(A)** over time. **(B)** Blocking CD11b using a blocking CD11b-mAb (10µg/ml) significantly increased infection of moDCs using HIV-C, while blocking CD11c with a blocking anti-human-CD11c-mAb resulted in significantly reduced productive infection of moDCs. Infection experiments were repeated at least three times in triplicates, differences were analyzed using GraphPad Prism and one-way ANOVA.
