# Supplementary material for: CR4 Signaling Contributes to a DC-Driven Enhanced Immune Response Against Complement-Opsonized HIV-1
Source: Front Immunol. 2020 Aug 14;11:2010. doi: 10.3389/fimmu.2020.02010 (PMC7457048; doi:10.3389/fimmu.2020.02010)
Supplement: Supplementary file 2 [file Presentation_1.PPTX]

## Slide 1
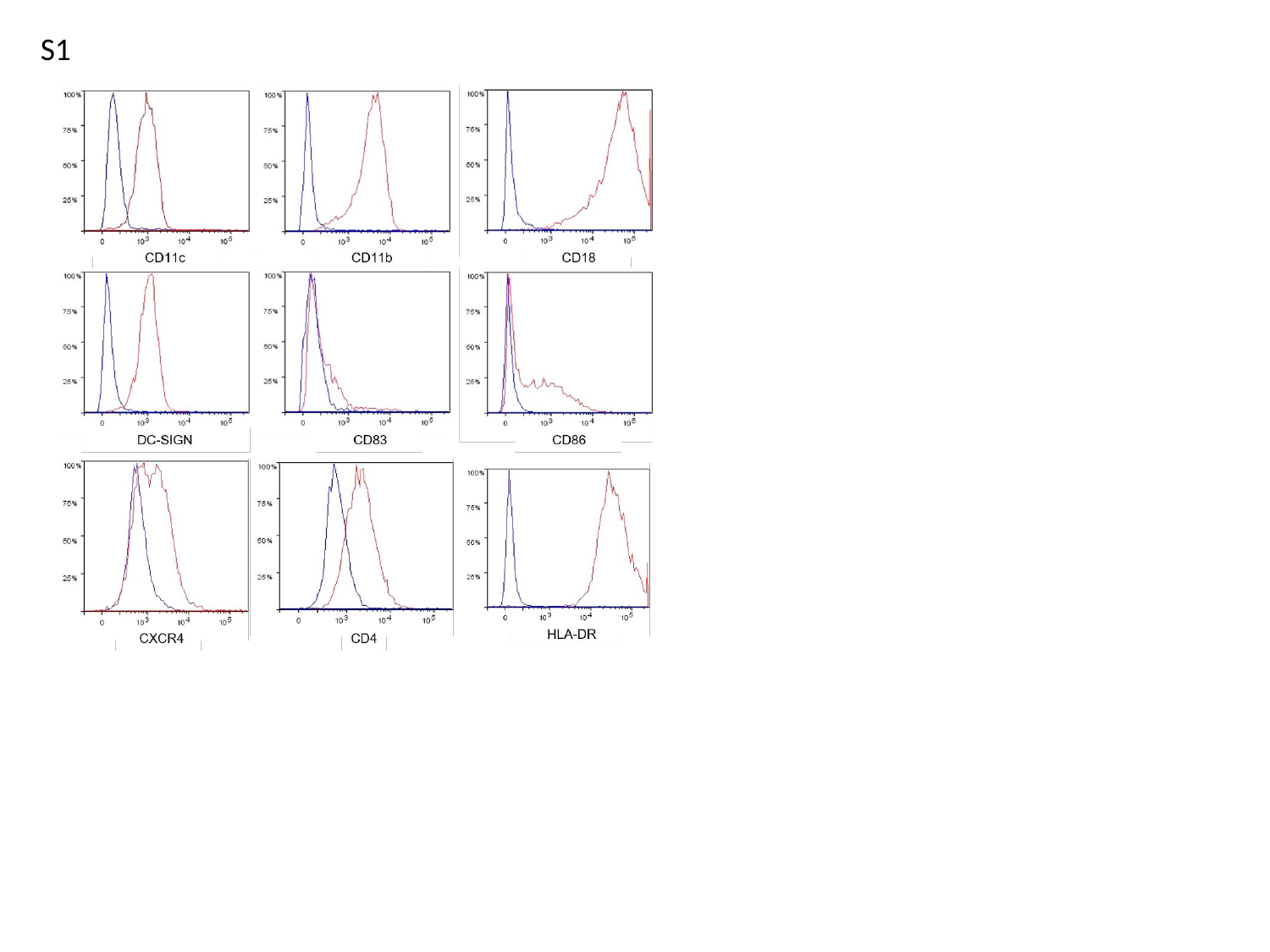

S1

## Slide 2
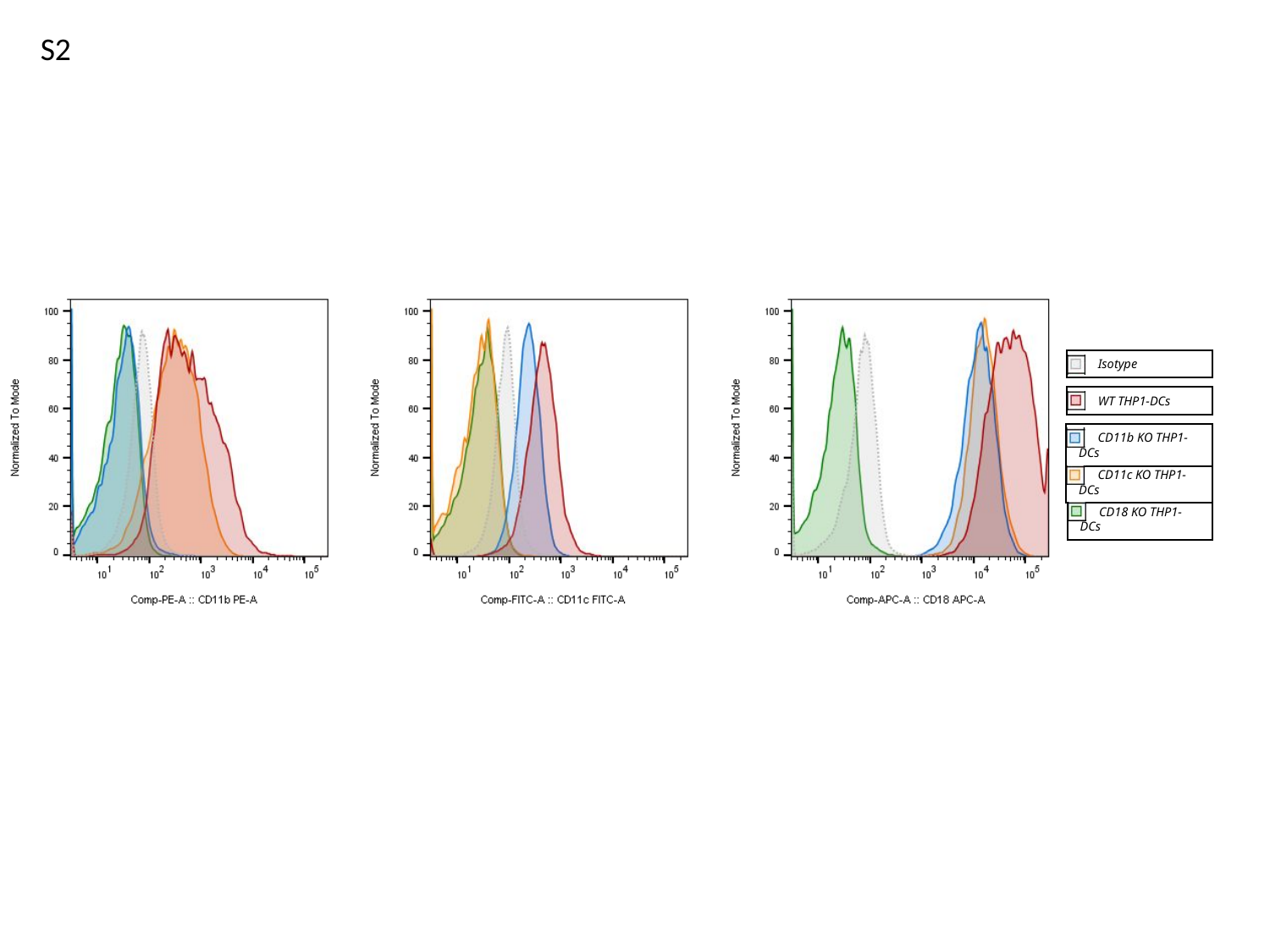

S2
 Isotype
 WT THP1-DCs
 CD11b KO THP1-DCs
 CD11c KO THP1-DCs
 CD18 KO THP1-DCs

## Slide 3
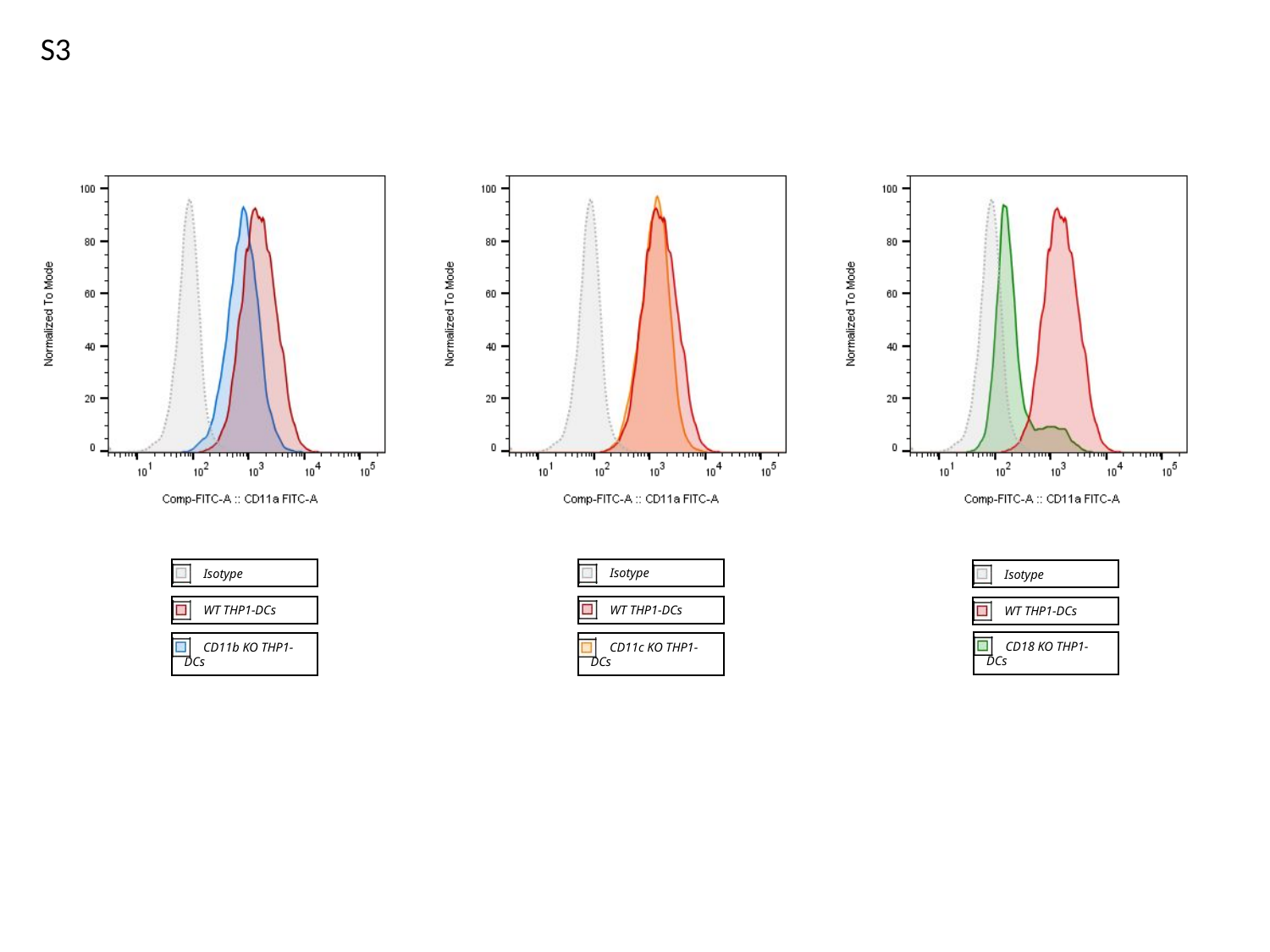

S3
 Isotype
 WT THP1-DCs
 CD11c KO THP1-DCs
 Isotype
 WT THP1-DCs
 CD11b KO THP1-DCs
 Isotype
 WT THP1-DCs
 CD18 KO THP1-DCs

## Slide 4
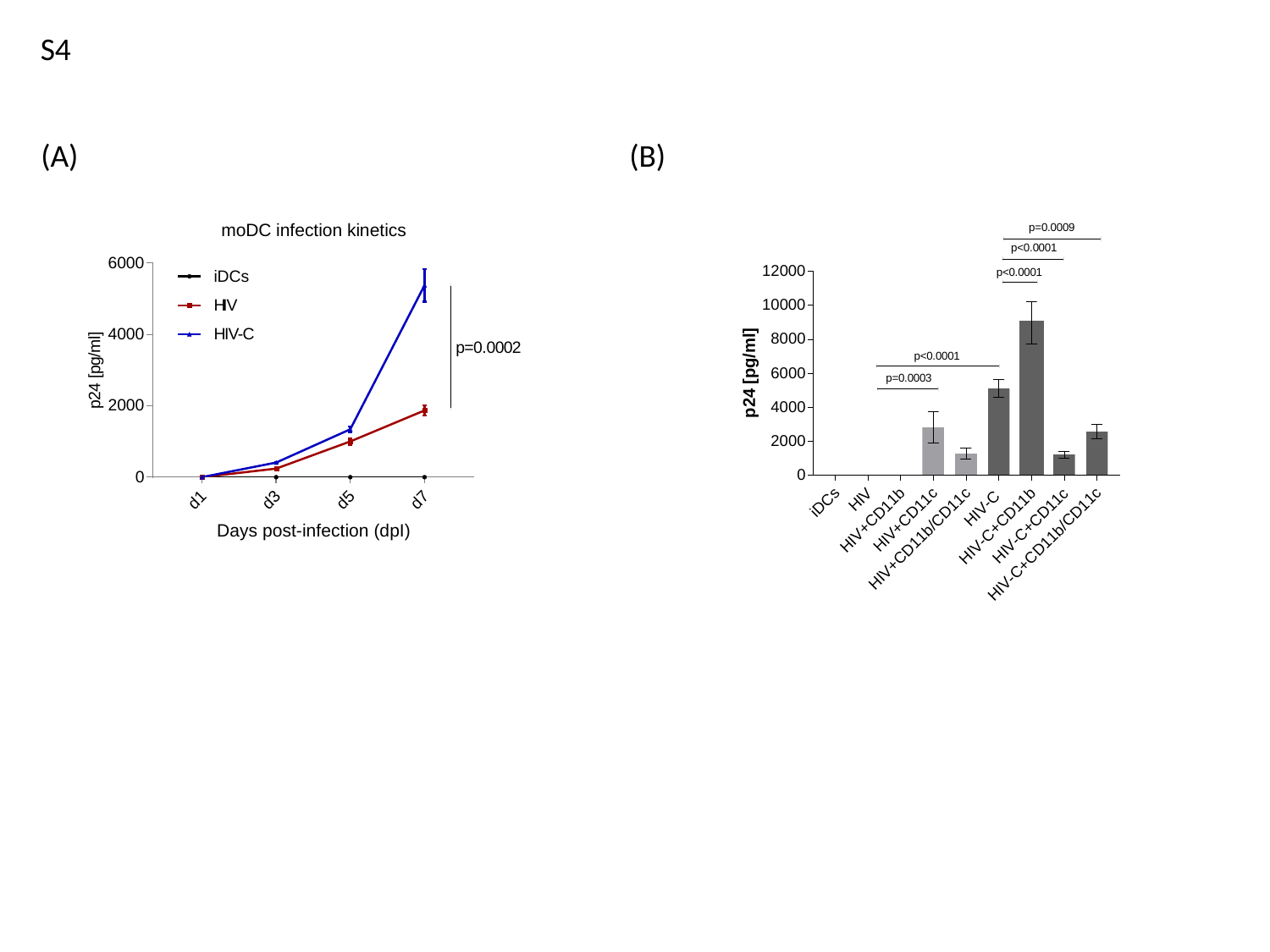

S4
(B)
(A)
